# Cucurbitacin I Reverses Tumor-Associated Macrophage Polarization to Affect Cancer Cell Metastasis

**DOI:** 10.3390/ijms242115920

**Published:** 2023-11-02

**Authors:** Xiaocheng Gong, Yunfei Liu, Keying Liang, Zixi Chen, Ke Ding, Li Qiu, Jinfen Wei, Hongli Du

**Affiliations:** School of Biology and Biological Engineering, South China University of Technology, University Town Campus, Guangzhou Higher Education Mega Centre, Panyu District, Guangzhou 510006, China; 202010108425@mail.scut.edu.cn (X.G.); 202120149279@mail.scut.edu.cn (Y.L.); 201910107991@mail.scut.edu.cn (K.L.); chenzixi2011@gmail.com (Z.C.); keding0302@gmail.com (K.D.); qiuli0531@gmail.com (L.Q.)

**Keywords:** cucurbitacin I, macrophage polarization, M2 macrophage, heme oxygenase-1 (*Hmox1*)

## Abstract

The tumor microenvironment plays a critical role in tumor progression and immune regulation. As one of the most important components of the tumor microenvironment, macrophages have become a new therapeutic target for inhibiting tumor progression. Despite the well-documented anticancer activity of cucurbitacin I, its effect on macrophages remains unclear. In this study, we established a coculture system of macrophages and cancer cells under hypoxic conditions to simulate the tumor-promoting environment mediated by M2-like macrophages. We determined whether cucurbitacin I modulates M2-like polarization in macrophages in vitro and conducted RNA sequencing to identify gene expression changes induced by cucurbitacin I in macrophages. The results indicated a remarkable inhibition of the M2-like polarization phenotype in macrophages following treatment with cucurbitacin I, which was accompanied by the significant downregulation of heme oxygenase-1. Moreover, we found that cucurbitacin I-treated macrophages reduced the migration of cancer cells by inhibiting the M2 polarization in vitro. These findings highlight the potential of cucurbitacin I as a therapeutic agent that targets M2-like macrophages to inhibit cancer cell metastasis. Our study provides novel insights into the intricate interplay among macrophage polarization, cucurbitacin I, and heme oxygenase-1, thereby opening new avenues for cancer treatment.

## 1. Introduction

Cancer continues to be a primary contributor to mortality and remains a significant global health concern. Despite advancements in cancer treatment, such as surgery, chemotherapy, and radiation therapy, the number of individuals affected by or succumbing to cancer remains high [1], and most treatments are accompanied by severe side effects [2,3,4]. The underlying reason for these limitations is the tumor microenvironment (TME), which hinders the efficacy of cancer drugs. The TME, which consists of stromal cells and extracellular matrix (ECM) components [5], is strongly immunosuppressive. The intricate interplay between these components facilitates tumor growth, metastasis, and invasion, thereby contributing to tumor escape from immune surveillance [6]. The efficacy of existing tumor-targeted drugs is often compromised due to the intricate composition and dynamics of the TME. Several studies have highlighted the challenges posed by the TME in hampering the efficacy of therapeutic interventions. For example, studies on certain targeted therapies, such as vemurafenib for BRAF-mutant melanoma, have demonstrated initial responses, but resistance mechanisms and the TME contributed to eventual treatment failure [7]. In addition, non-small cell lung cancer often develops resistance to EGFR inhibitors, such as erlotinib and gefitinib, primarily because of mutations and alterations in the TME. These factors collectively curtail the effectiveness of treatment [8] and highlight how the TME’s intricate interactions and adaptability contribute to treatment failure. They also emphasize the need for a holistic approach that considers the interplay between tumor cells and their surrounding milieu. Thus, tumor cell-centric therapies are being replaced by TME-centric therapies.

Macrophages are important components of the TME that exert a significant influence on both tumor progression and responses to therapy [9]. Among the cell types associated with the TME, tumor-associated macrophages (TAMs) and their precursors represent the predominant fraction of myeloid infiltrates in most human solid malignancies [10]. Within the TME, macrophages exhibit remarkable plasticity and can change phenotypes, including M1- and M2-like polarization. M1-like macrophages exhibit antitumor characteristics by fostering inflammation and immune activation. Conversely, M2-like macrophages exhibit protumor properties, which contribute to immune suppression and tissue remodeling [11,12]. TAMs are commonly found in diverse cancer types and are linked to tumor progression [13,14,15,16], angiogenesis, metastasis, and drug resistance [17]. TAMs can be recruited and manipulated by tumor-derived signals, such as cytokines, chemokines, and growth factors, to adopt an M2-like phenotype [18]. In this state, TAMs promote tumor growth by secreting immunosuppressive factors, facilitating angiogenesis, remodeling the extracellular matrix, and suppressing the antitumor immune response. Thus, there is a growing emphasis on developing targeted therapies aimed at reprogramming their function. Regulating TAMs, especially repolarizing them from the M2-like phenotype to the pro-inflammatory M1-like phenotype, has emerged as a promising strategy in cancer treatment.

In the current landscape of cancer treatment, there are various techniques and methods aimed at reprogramming TAMs from the M2 type to the M1 type to enhance anti-cancer immune responses. These methods encompass immune checkpoint inhibitors, CAR-T cell therapy [19], cytokine therapy, RNA interference, and targeted drug therapy, among others. Immune checkpoint inhibitors like PD-1 and CTLA-4 antibodies [20], as well as CAR-T cell therapy, enhance their anti-tumor aggressiveness by activating the host immune system and inducing a transformation of M2-polarized macrophages into the M1 type. Although these methods exhibit outstanding efficacy in certain scenarios, they are also accompanied by immune-related toxicities and adverse reactions. Cytokine therapy, including interferon-γ, tumor necrosis factor α, and interleukin-12, has the direct capability to influence the polarization state of TAMs, prompting their shift towards M1-type macrophages [21]. Nonetheless, these therapies may lead to cytokine release syndrome and other discomforts. RNA interference and CRISPR-Cas9 [22] techniques offer the potential to selectively alter the expression of specific genes in TAMs, facilitating the conversion from M2 to M1 type. However, they come with challenges related to delivery and safety. Some drugs, such as PI3K inhibitors and CSF1R inhibitors [23,24,25], have the capacity to interfere with M2 polarization in TAMs, inducing them to shift to the M1 type. These drugs can be employed in clinical treatment to intervene in the tumor microenvironment and enhance treatment outcomes. Besides these conventional techniques, emerging therapies are gradually demonstrating their ability to reprogram macrophages. These include engineered macrophages based on CAR technology like “MacTrigger” [26], CAR-M technology [27], and exosome technology derived from M1-type macrophages [28]. These therapies, to some extent, induce a phenotypic transformation of TAMs from cancer patients into macrophages with an M1-like phenotype. Nevertheless, targeted drug therapy still appears to maintain its unique advantages. It is comparatively more feasible to achieve precise targeting and it is associated with lower research and treatment costs. Additionally, targeted drugs are typically more readily deliverable to tumor sites through various delivery systems, thus providing expanded treatment opportunities. Although various techniques and approaches have their limitations, targeted drug therapy has demonstrated potential and relatively lower risks in polarizing TAMs within the tumor microenvironment. Therefore, targeted drug therapy may emerge as a promising approach in future cancer treatments.

Currently, research on drugs that repolarize TAMs into tumor suppressor phenotypes remains in an early phase of therapy. Therefore, more in-depth studies are needed for further expansion and insight in the future [29]. Because of the low cost and sufficient proportion of data, “new uses for old drugs” has gained interest as an important option for cancer therapy [30,31]. Cucurbitacins are a class of tetracyclic triterpenoid compounds derived from plants of the Cucurbitaceae and Brassicaceae families as well as other families. Of these compounds, cucurbitacin I (CuI) is a subtype within the cucurbitacin family. Recently, there has been a surge in the exploration of the anticancer properties of cucurbitacin I. This natural compound exhibits cytotoxicity against a variety of cancer types, including lung cancer, glioblastoma, and other malignancies. The antitumor mechanisms of cucurbitacin I involve modulating cell cycle progression, inducing apoptosis, and inhibiting cancer cell migration and invasion. Thus, cucurbitacin I is a promising candidate for anticancer therapy. However, our understanding of the effect of cucurbitacin I on TAMs and the intricate molecular mechanisms governing its influence on macrophage behavior is limited. Furthermore, assessing its potential as a therapeutic agent for cancer is necessary. In this study, we examined the antitumor and antimetastatic effects of cucurbitacin I in breast and colorectal cancer cells and explored the potential mechanism of its effect on polarization changes in TAMs.

## 2. Results

### 2.1. Cucurbitacin I Exerts Antitumorigenic Effects on Breast and Colorectal Cancer Cells

**Figure 1:**
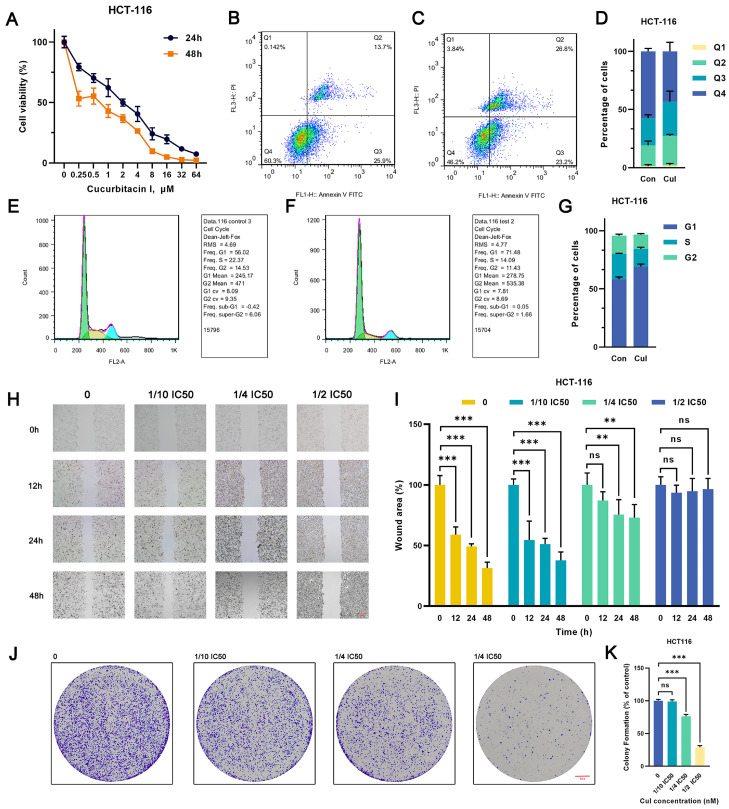
Cucurbitacin I has an inhibitory effect on the CRC cell phenotype. (**A**) HCT116 cells were treated with cucurbitacin I (0, 0.25, 0.5, 1, 2, 4, 8, 16, 32, 64 μM) for 24 and 48 h, and cell viability (%) was measured. (**B**–**D**) Representative flow cytometry plots and quantitative results (**D**) depicting the apoptotic characteristics of HCT116 cells treated with cucurbitacin I (500 nM) (**C**) compared with non-treated ones (**B**). (**E**–**G**) Flow cytometry indicated that cucurbitacin I resulted in changes in cell proportions at the G1/S phase in HCT116 cells cultured in the absence (**E**) and presence (**F**) of cucurbitacin I (500 nM). In the flow cytometry plot, the green peak represents cells in the G1 phase, the yellow peak represents cells in the S phase, and the blue peak represents cells in the G2 phase. (**H**,**I**) Scratch assay of HCT116 cells treated with cucurbitacin I at different concentrations (0 nM, 1/10, 1/4, 1/2 IC50), and the resulting wound area (%) was calculated in (**I**). Scale bar represents 100 μm. (**J**,**K**) Colony formation assay of HCT116 cells treated with cucurbitacin I at different concentrations (0 nM, 1/10, 1/4, 1/2 IC50), and the colony numbers are shown in (**K**). Scale bar represents 5 mm. The values presented are expressed as mean ± standard deviation (SD) and are based on data obtained from three biologically independent samples. No significance: ns, *p* < 0.01: **, *p* < 0.001: ***.

Various concentrations of cucurbitacin I (0, 0.25, 0.5, 1, 2, 4, 8, 16, 32, and 64 μM) significantly suppressed the viability of MCF7 and HCT116 cells, exhibiting a pronounced concentration gradient and time-dependent inhibition effect (Figure 1A and Figure S1A). The 50% inhibitory concentrations (IC50) of cucurbitacin I in HCT116 cells were 2.028 μM (24 h) and 0.7184 μM (48 h), whereas those in MCF7 cells were 8.172 μM (24 h) and 1.195 μM (48 h). Subsequently, we used cucurbitacin I concentrations of 1/10 IC50, 1/4 IC50, and 1/2 IC50 for in vitro studies. Of note, cucurbitacin I significantly reduced colony formation and inhibited cell migration in MCF7 and HCT116 cells, indicating its capability to substantially curb the autonomous survival and migratory potential of tumor cells (Figure 1H–K and Figure S1H–K). Next, we determined whether the proliferation inhibition mediated by cucurbitacin I resulted from cell cycle arrest, apoptosis induction, or a combination of both. As shown in Figure 1B–G and Figure S1B–G, treatment with cucurbitacin I resulted in an increase in early apoptotic cells (Q2 quadrant). Furthermore, there was a significant increase in the proportion of cells in the G1 phase, coupled with a reduction in the percentages of cells in the S phase in both MCF7 and HCT116 cell populations. Overall, cucurbitacin I exerted its anticancer effects by inhibiting cell proliferation, arresting cell cycle progression, and inducing apoptosis.

### 2.2. Induction of M2 Polarization in Macrophages by Coculturing with Cancer Cells and Hypoxia

**Figure 2:**
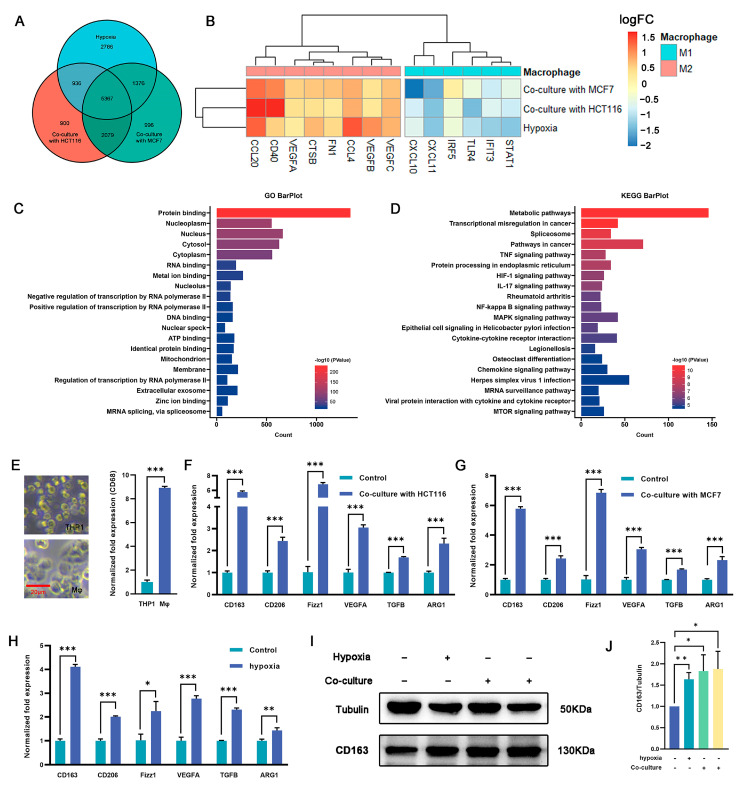
Regulation of macrophage polarization and expression of M2-type markers by culturing macrophages under hypoxia or in a coculture system. (**A**) Venn diagram showing the overlap of DEGs among the three macrophage treatment groups (hypoxia, cocultured with HCT116, or cocultured with MCF7 cells) (*n* = 3 samples) in comparison with THP-1 derived macrophages. (**B**) Expression (log2FC) of selected M1- and M2-related genes in the three treated macrophage groups, (A) represented as a heatmap. Colors indicate group identity: blue, M1-related genes; red, M2-related genes; adjusted *p* ≤ 0.05. (**C**,**D**) GO and KEGG analyses showing the top 20 functional annotations of the upregulated overlapping DEGs from (**A**), adjusted *p* ≤ 0.05. (**E**) THP1 was induced by PMA for 48 h and exhibited typical M0 macrophage morphology, as represented by bright-field images and upregulated expression of CD163 as measured via qRT-PCR. Scale bar represents 20 μm. (**F**,**G**) M2-related genes (*Cd163*, *Cd206*, *Fizz1*, *Vegfa*, *Tgfβ*, and *Arg1*) were significantly upregulated in macrophages cocultured with HCT116 cells (**F**) and MCF7 cells (**G**). (**H**) qRT-PCR analysis revealed upregulation of M2-related gene expression in the hypoxia group vs. controls (namely, normoxia). (**I**,**J**) CD163 protein expression in macrophage cells was detected under hypoxia (the second protein lane) or in a coculture system with MCF7 (the third protein lane) or HCT116 (the fourth protein lane) cells versus the control group (the first protein lane) without dual treatment, as determined by Western blot analysis. Tubulin was used for normalization. The values presented are expressed as mean ± standard deviation (SD) and are based on data obtained from three biologically independent samples. *p* < 0.05: *, *p* < 0.01: **, *p* < 0.001: ***.

Macrophages play an important role in the immune response and tissue homeostasis by demonstrating remarkable plasticity in response to microenvironmental cues. In this study, we examined whether cocultivation and exposure to hypoxia influenced the polarization of macrophages toward the M2 phenotype. THP1 monocytes were treated with PMA (100 ng/mL) for 48 h to induce macrophage differentiation (Figure 2E), followed by cultivation under hypoxia or coculture conditions. RNA-seq analysis was used to identify differentially expressed genes (DEGs) between macrophages subjected to coculture or hypoxia treatment and untreated macrophages (Figure S2A–D). The intersection of these three DEG sets was examined to elucidate shared differences (Figure 2A). The RNA-seq analysis revealed a total of 5367 DEGs, of which 2325 were upregulated across all three experimental groups and 2464 were downregulated (Figure S2E). Of note, following coculture with tumor cells or exposure to hypoxia, some genes associated with the M2 phenotype were upregulated, whereas others related to the M1 phenotype were downregulated (Figure 2B). Gene ontology (GO) and Kyoto Encyclopedia of Genes and Genomes (KEGG) enrichment analyses were performed on the DEGs, and distinct pathways associated with differential expression were identified. The findings depicted in Figure 2C,D and Figure S2F,G indicate that the upregulated genes exhibited significant enrichment in various pathways, including but not limited to the metabolic pathway, pathways in cancer, HIF-1 signaling pathway, IL-17 signaling pathway, NF-Kappa B signaling pathway, and MAPK signaling pathway. Conversely, the downregulated genes showed significant enrichment in pathways such as the metabolic pathway, calcium signaling pathway, NOD-like receptor signaling pathway, and carbon metabolism. These findings suggest that coculture with tumor cells and hypoxic conditions promote macrophage polarization toward an M2-like phenotype.

Validation was carried out using qRT-PCR, which revealed a 2.1–3.2-fold (*p* < 0.05) increase in the expression of the key M2 marker *Cd163* in macrophages subjected to coculture and hypoxia exposure compared with the controls. Similarly, the expression of *Cd206*, a well-established M2 surface marker, was also significantly increased by 1–4.2-fold in the coculture and hypoxia groups (*p* < 0.05). Furthermore, the expression of transforming growth factor beta (*Tgfβ*), arginase 1 (*Arg1*), resistin-like beta (*Fizz1*), and vascular endothelial growth factor A (*Vegfa*), which are potent anti-inflammatory cytokines associated with the M2 phenotype, were markedly upregulated by 0.6–6.1-fold in cocultured and hypoxia-exposed macrophages (*p* < 0.05) (Figure 2F–H). To validate the qRT-PCR results, Western blotting (WB) analysis was performed to measure the expression of CD163 protein. Consistent with the qRT-PCR results, the protein levels exhibited a significant increase, ranging from 0.6–0.9-fold, in both the coculture and hypoxia groups compared with the control group (*p* < 0.05) (Figure 2I,J). These results suggest that the combination of coculture and hypoxia effectively induces M2 polarization in macrophages.

### 2.3. Cucurbitacin I Inhibits M2-Like Polarization of Macrophages

**Figure 3:**
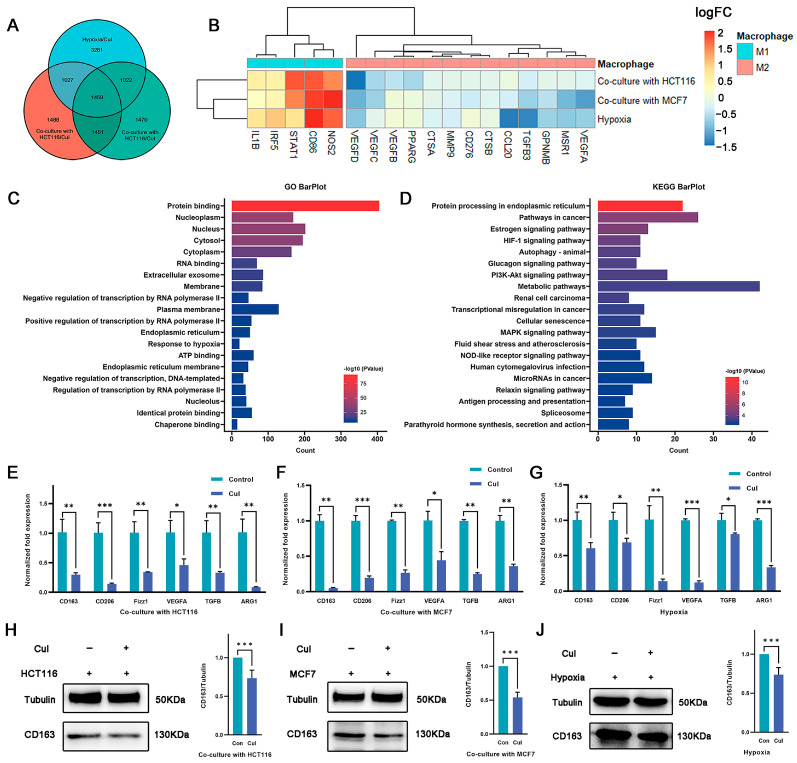
Macrophage polarization is reversed by cucurbitacin I under hypoxia or coculture conditions. (**A**) Venn diagram representing the overlap of DEGs among the three macrophage treatment groups (under hypoxia and cucurbitacin I treatment, cocultured with HCT116 or MCF7 cells and cucurbitacin I treatment) compared to corresponding groups without cucurbitacin I treatment. (**B**) Expression (log2FC) of selected M1- and M2-related genes in the three macrophage treatment groups, (**A**) represented as a heatmap. Colors indicate group identity: blue, M1-related genes; red, M2-related genes; adjusted *p* ≤ 0.05. (**C**,**D**) GO and KEGG analyses showing the top 20 functional annotations of the downregulated overlapping DEGs from (**A**). Adjusted *p* ≤ 0.05. (**E**–**G**) Normalized fold expression of M2-related gene in macrophages after cucurbitacin I treatment (300 nM) compared with the untreated controls either cocultured with HCT116 cells (**E**), cocultured with MCF7 cells (**F**), or under hypoxia (**G**). (**H**–**J**) Cucurbitacin I (300 nM) downregulated the protein levels of CD163 in macrophages, cultured in three systems: cocultured with HCT116 cells (**H**), cocultured with MCF7 cells (**I**), or under hypoxia (**J**). The values presented are expressed as mean ± standard deviation (SD) and are based on data obtained from three biologically independent samples. *p* < 0.05: *, *p* < 0.01: **, *p* < 0.001: ***.

Although cucurbitacin I exerts antitumor effects by inhibiting tumor cell growth, its potential impact on macrophage polarization is unknown. In this study, cucurbitacin I-treated macrophages were under coculture with tumor cells as well as hypoxia conditions. RNA-seq analysis was performed to identify DEGs between cucurbitacin I-treated and untreated macrophages (Figure S3A–D). The intersection of three DEG sets was used to identify shared characteristics (Figure 3A). In total, 1459 intersecting genes were identified, of which 592 were downregulated and 563 were upregulated across all three experimental groups (Figure S3E). Of note, after cucurbitacin I treatment, the sequencing data indicated a downregulation of genes associated with the M2 phenotype, whereas other genes linked to the M1 phenotype were upregulated (Figure 3B). GO and KEGG enrichment analyses were conducted on the DEGs. As shown in Figure 3C,D and Figure S3F,G, the downregulated genes exhibited significant enrichment in pathways, including pathways in cancer, HIF-1 signaling pathway, autophagy, PI3K–Akt signaling pathway, and transcriptional mis-regulation in cancer. Conversely, the upregulated genes exhibited significant enrichment in metabolic pathway, thermogenesis, carbon metabolism, and amino acid biosynthesis.

Because the RNA-seq results revealed the anticancer properties and immunomodulatory effects of cucurbitacin I on macrophages, we determined the effect of cucurbitacin I on macrophage polarization under hypoxia and in a coculture system. qRT-PCR analysis revealed a decrease in the expression of M2 markers and M2-associated cytokines, such as *Cd206*, *Cd163*, and *Arg1*, following cucurbitacin I treatment (Figure 3E–G). We quantitated the relative levels of CD163 protein using densitometry analysis. Western blot analysis revealed a marked reduction in CD163 protein following drug treatment (Figure 3H–J). Comparatively, cucurbitacin I treatment resulted in a significant upregulation of M1-associated factors at the transcriptional level. For example, inducible nitric oxide synthase (*iNOS*), tumor necrosis factor (*Tnf*), C–X–C motif chemokine ligand 2 (*Cxcl2*), and interleukin-1 beta (*IL-1β*) mRNA levels were markedly increased compared with the control group (Figure S3E–G). These results suggest that cucurbitacin I inhibits the shift of macrophages toward the M2 phenotype by regulating the expression of M1- and M2-associated genes and cytokines. Further studies are needed to identify the precise mechanisms responsible for the inhibitory effects of cucurbitacin I on M2 polarization.

### 2.4. Hmox1 Was Significantly Downregulated in M2-Like Macrophage under Cucurbitacin I

**Figure 4:**
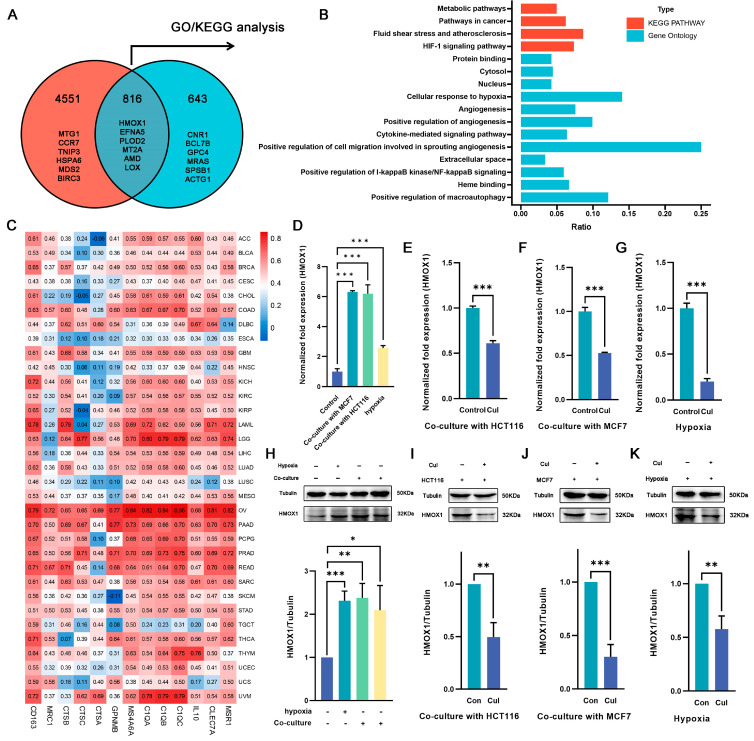
Cucurbitacin I downregulates the expression of the M2-related gene *Hmox1* in macrophages from hypoxia or coculture systems. (**A**) Overlap of DEGs at two intersections, presented as a Venn diagram. (**B**) A statistical histogram showing several *Hmox1*-enriched GO and KEGG pathways. (**C**) Heatmap depicting *Hmox1* as being significantly linked with the expression of M2-related genes in 33 TCGA cancer types. (**D**–**H**) The mRNA and protein levels of *Hmox1* were significantly elevated in macrophages either in coculture systems with MCF7 (the third protein lane) or HCT116 (the fourth protein lane) cells, or under hypoxia (the second protein lane) via qRT-PCR and Western blot analysis. (**E**–**G**,**I**–**K**) The mRNA and protein levels of *Hmox1* were remarkably decreased in macrophages treated with cucurbitacin I either in cocultured systems with HCT116 or MCF7 cells or under hypoxia via qRT-PCR and Western blot analysis. Results for all panels were independently reproduced at least three times. The values presented are expressed as mean ± standard deviation (SD) and are based on data obtained from three biologically independent samples. *p* < 0.05: *, *p* < 0.01: **, *p* < 0.001: ***.

To elucidate the specific mechanism by which cucurbitacin I regulates macrophage polarization, we selected genes that exhibited changes under both hypoxia and coculture conditions and following cucurbitacin I treatment. In total, 816 DEGs were identified (Figure 4A). Of these, 359 were upregulated after hypoxia/coculture and downregulated after cucurbitacin I treatment, whereas 264 were downregulated after hypoxia/coculture and upregulated after cucurbitacin I treatment. We hypothesized that these 816 DEGs may be associated with the mechanism through which cucurbitacin I inhibits macrophage M2 polarization. Consequently, we performed GO and KEGG pathway enrichment analyses. The results of our sequencing revealed that, among the 816 DEGs, *Hmxo1* exhibited significant enrichment across multiple pathways, including the HIF-1 signaling pathway, cellular response to hypoxia, angiogenesis, cytokine-mediated signaling pathway, extracellular space, heme binding, and positive regulation of macroautophage (Figure 4B). This enrichment pattern suggests that *Hmxo1* might play an important role in mediating the inhibitory effects of cucurbitacin I on M2 macrophage polarization. To determine the correlation of *Hmxo1* and M2-like macrophages, the correlation coefficient of *Hmox1* with M2 marker genes was calculated using The Cancer Genome Atlas (TCGA) dataset containing 33 cancer types (Figure 4C). Interestingly, the analysis revealed positive correlations between *Hmox1* and a number of established M2 macrophage markers across multiple cancer types. This correlation pattern suggests the involvement of *Hmox1* in driving the M2-like polarization of macrophages within the intricate TME. In addition, our RNA-seq data consistently revealed a marked upregulation of *Hmox1* expression under coculture or hypoxic culture conditions, followed by a significant downregulation upon treatment with cucurbitacin I. These dynamic expression changes underscore the responsiveness of *Hmox1* to external stimuli and its potential involvement in modulating macrophage polarization. To validate these findings at the transcriptional and protein levels, we performed qRT-PCR and WB analyses to quantify the expression of *Hmox1* (Figure 4D–K). The results supported the observed dynamic regulation of *Hmox1* expression under coculture, hypoxic conditions, and cucurbitacin I treatment, providing mechanistic insights into the regulation of *Hmox1* in macrophages.

**Figure 5:**
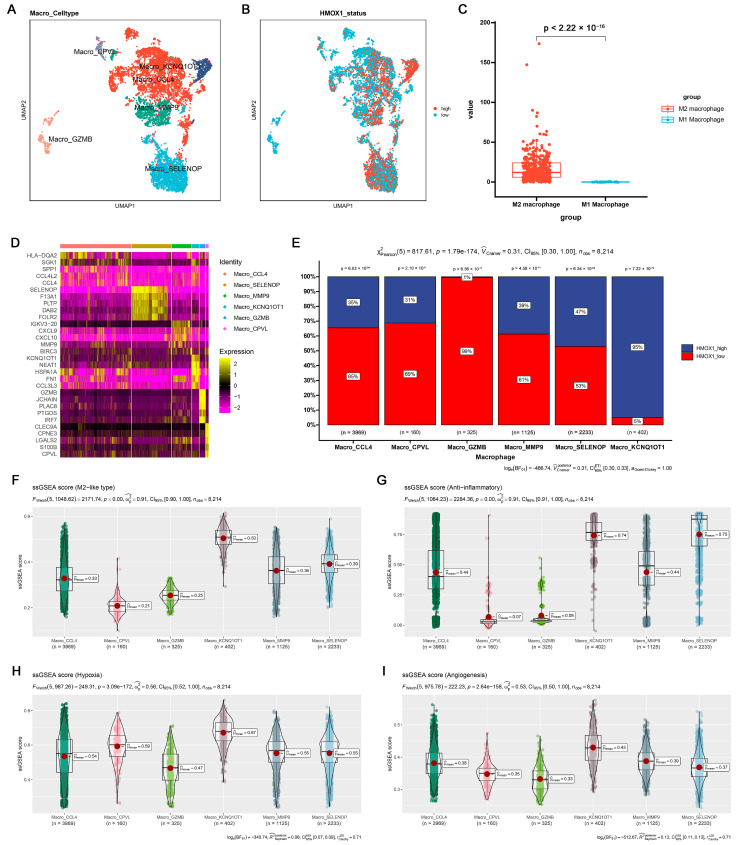
Single-cell analysis of the *Hmox1* expression profile revealed a relationship between Hmox1 and cancer-related processes in BRCA. (**A**) The UMAP visualization of macrophages from patients with breast cancer, showing the formation of 6 main clusters shown in different colors. The functional description of each cluster was determined by the gene expression characteristics of each cluster. (**B**) The UMAP visualization of macrophages, with each cell colored based on the relative normalized expression levels of Hmox1. (**C**) Expression levels of Hmox1 in M2 macrophages in comparison with M1 macrophages from BRCA tumor samples; boxes show the median and whiskers indicate the 95th and 5th percentiles. (**D**) Heatmap of marker gene expression (y-axis) across BRCA macrophages clusters identified (x-axis). (**E**) Histogram displaying the cell percentage in the high and low Hmox1 expression groups in five macrophage subclusters. (**F**–**I**) Boxplot displaying ssGSEA scores in seven macrophage subclusters from scRNA-seq of BRCA. The ssGSEA score associated with the M2-like type (**F**), anti-inflammatory properties (**G**), hypoxia (**H**), and angiogenesis (**I**); boxes show the median and whiskers indicate the 95th and 5th percentiles.

To further examine the potential relationship among inflammation, *Hmox1* expression, and macrophage M2 polarization, single-cell tumor transcriptome datasets of breast and colorectal cancer cohorts were analyzed. Initially, the cells were classified into distinct clusters based on specific marker expression patterns (Figures S4A and S6A). For example, T/NK cells were characterized by the presence of *Cd2* and *Cd3d* markers, whereas myeloid cells were characterized by the expression of *Cd14* and *Cd68* markers (Figures S4B and S6B). Interestingly, the analysis revealed that *Hmox1* exhibited remarkable specificity for myeloid cells, predominantly expressed within this cell population, whereas it exhibited minimal to negligible expression in other cell types within the TME of both breast and colorectal cancers (Figures S4D and S6D). Furthermore, within the myeloid cell population, we further divided the cells into monocytes and macrophages based on the expression of other markers (Figures S4C and S6C). To gain further insights into the role of *Hmox1*, macrophages were isolated from the samples and further separated into several new clusters (Figure 5A,D and Figure S5A,D). In addition, macrophages were divided into high and low *Hmox1* expression groups, and the distribution of these groups within different macrophage clusters was analyzed (Figure 5B and Figure S5B). Interestingly, the analysis indicated that the M2 macrophage cluster exhibited higher levels of *Hmox1* expression than the M1 macrophage cluster (Figure 5C and Figure S5C). The single-sample gene set enrichment analysis (ssGSEA) scores for various gene sets, including those associated with the M2-like type, anti-inflammatory properties, and immune escape, were calculated to determine the characteristics of various macrophage subsets. The results indicated that Macro_KCNQ10T1 and Macro_SPP1 exhibited high M2-like and anti-inflammatory scores in breast cancer and colorectal cancer, respectively (Figure 5F–I, Figures S4E–H, S5F–I and S6E–H). Consistent with these results, the M2 macrophage cluster, including Macro_KCNQ10T1 and Macro_SPP1, exhibited a higher proportion of *Hmox1*-high-expression macrophages (Figure 5E and Figure S5E). This finding suggests a regulatory role of HMOX1 in driving macrophage polarization toward the M2 phenotype.

### 2.5. Hemin Restored the Downregulation of Hmox1 in Macrophages Caused by Cucurbitacin I and the Inhibition of Tumor Cell Migration

**Figure 6:**
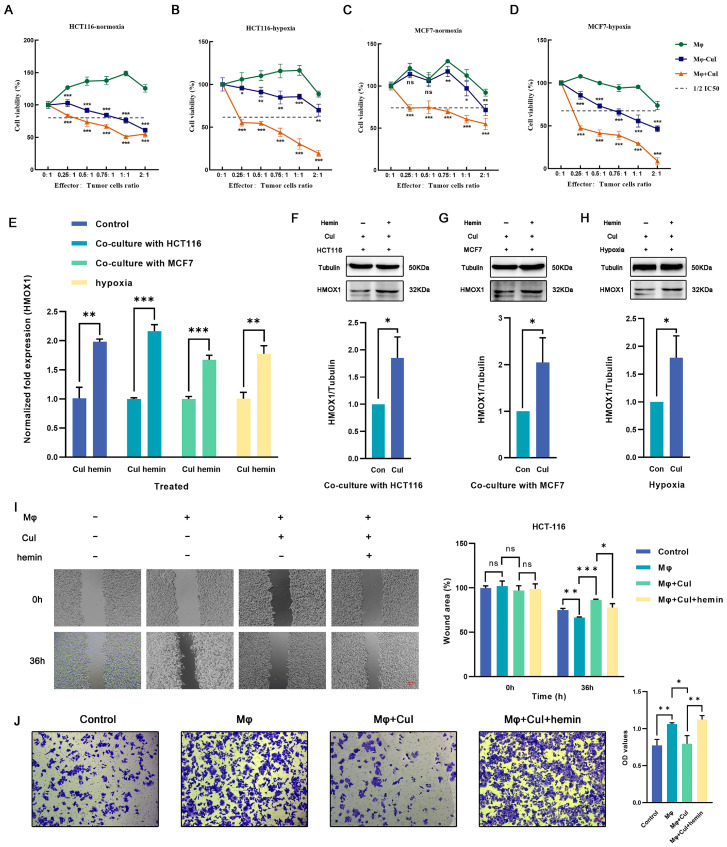
Cucurbitacin I inhibits M2 polarization of macrophages by decreasing the expression of *Hmox1* and augments the tumoricidal activity, which can be reversed by hemin. (**A**–**D**) Cell viability (%) of HCT116 cells and MCF7 cells was assayed, including those cocultured with macrophages alone, with macrophages after being treated with cucurbitacin I, or in a triple coculture system including macrophages and cucurbitacin I under normoxic or hypoxic conditions. (**E**) Hemin reversed the mRNA level of *HMOX1* downregulated by cucurbitacin I cocultured with MCF7 cells, cocultured with HCT116 cells, or under hypoxia. (**F**–**H**) Hemin restored the protein levels of HMOX1 downregulated by cucurbitacin I, either in a coculture system with HCT116 cells (**F**), in a coculture system with MCF7 cells (**G**), or under hypoxia (**H**). (**I**,**J**) Cell migration and invasion capacity of HCT116 alone or cocultured with macrophages (alone, cucurbitacin I-treated macrophages, or both cucurbitacin I and hemin-treated macrophages), as determined by a wound-healing assay and Transwell coculture system, respectively. The concentration of hemin is 1 μM, and the concentration of cucurbitacin I is 500 nM. Scale bar represents 100 μm (**I**) and 50 μm (**J**). The values presented are expressed as the mean ± standard deviation (SD) and are based on data obtained from three biologically independent samples. No significance: ns, *p* < 0.05: *, *p* < 0.01: **, *p* < 0.001: ***.

The cytotoxic effects of cucurbitacin I on tumor cells divided into three groups (a coculture group of untreated macrophages with tumor cells, a coculture group of cucurbitacin I-treated macrophages with tumor cells, and a coculture group of cucurbitacin I, macrophages, and tumor cells) were assessed. Under low effector-to-target ratios, the control group showed no significant cytotoxicity against tumor cells; however, macrophages treated with cucurbitacin I exhibited significant inhibition of tumor cell growth (Figure 6A–D). To further substantiate the potential of cucurbitacin I in suppressing macrophage M2 polarization and, consequently, inhibiting the tumor-promoting effects mediated by M2 macrophages, we conducted a series of functional assays. Transwell migration and scratch assays were used to assess how cucurbitacin I treatment influences the migration and invasion capabilities of macrophages. The results indicated robust inhibition of tumor cell migration following exposure to cucurbitacin I-treated macrophages. In the Transwell migration assay, cucurbitacin I-treated macrophages exhibited a marked reduction in tumor cell migration. The quantitation of migrating tumor cells revealed a significant decrease compared with the control group, suggesting a potent inhibitory effect of cucurbitacin I (Figure 6J and Figure S7B). Similarly, in a scratch assay, cucurbitacin I treatment resulted in a marked inhibition of tumor cell migration. The wound closure analysis revealed a substantial reduction in the closure rate of the scratch in the presence of cucurbitacin I-treated macrophages, further supporting the notion of a suppressive effect of cucurbitacin I on macrophage-mediated tumor cell migration (Figure 6I and Figure S7A).

Meanwhile, we conducted additional experiments by introducing hemin, a well-known inducer of *Hmox1* expression (Figure 6E–H). Interestingly, upon *Hmox1* upregulation induced by hemin, we observed a significant reduction in the inhibitory effects of cucurbitacin I on macrophage-mediated tumor cell migration and invasion (Figure 6I,J and Figure S7A,B). These findings provide evidence for the inhibitory effects of cucurbitacin I on macrophage-mediated tumor cell migration and invasion. A notable reduction in migration observed in both Transwell migration and scratch assays underscores the potential of cucurbitacin I as a modulator of macrophage polarization and its ability to counteract the protumor properties of M2 macrophages.

## 3. Discussion

In the field of cancer research, exploration of natural compounds holds promising prospects. As a candidate drug, cucurbitacin I exhibits potent cytotoxic effects against various tumor types. Several studies have indicated that cucurbitacin I partially inhibits diverse phenotypes of several cancers, such as lung [32,33], gastric [34], and ovarian cancers. The present study affirmed the cytotoxicity of cucurbitacin I in breast and colorectal cancer cell lines. It also suppressed colony formation and inhibited cell migration, which is consistent with previous studies. Cucurbitacin I can impede cell cycle progression and induce apoptosis through multiple pathways in various cancer cell lines. For example, it induces apoptosis in ovarian cancer cells through oxidative stress and the p190B-Rac1 signaling pathway [35]. Furthermore, it causes G2/M phase arrest in gastric cancer cells by disrupting redox homeostasis [34]. Similarly, we demonstrated that cucurbitacin I induces cell cycle arrest and apoptosis in breast and colorectal cancer cell lines, further supporting the multifunctional potential of cucurbitacin I in treating diverse tumor types. Although the cytotoxic effects of cucurbitacin I on various cancer cells have been extensively documented, its impact on TAMs remains unknown.

The TME is of profound importance to tumor therapy. It exerts an important role in various aspects of tumor progression and treatment response [6,36,37,38,39]. It represents a complex network comprising diverse cellular and noncellular components that interact dynamically with tumor cells. Hypoxia, which is a common characteristic of solid tumors, has been reported to reduce the efficacy of radiation therapy and some chemotherapeutic agents [40,41,42,43]. Because of the intricacies of the TME, characterized by cellular heterogeneity and hypoxic conditions, we used hypoxic culture and coculture of tumor cells with macrophages to simulate the in vivo TME. This design simulated the dynamic interactions and hypoxic environment observed within tumors, which we used to decipher the underlying molecular mechanisms and functional implications associated with the diverse cellular components and oxygen-deprived nature of the TME. The results indicated that coculture with tumor cells and culture under hypoxic conditions enhanced the M2 polarization of macrophages in vitro. This discovery is consistent with previously reported research results showing that class M2 macrophages can enhance proliferation, angiogenesis, EMT, migration, and invasion in various cancers [44,45,46,47].

In addition to evaluating the anticancer potential of cucurbitacin I, we also determined whether it enhanced the antitumor immune response by modulating the activation and function of immune cells and shaping the immune landscape through cytokine secretion. In contrast to previous studies, we provided a more nuanced understanding of cucurbitacin I’s dual role: its ability to not only suppress tumor cell phenotypes but also inhibit the M2 polarization of macrophages in vitro. Typically, macrophages exhibit two distinct polarization states, including M1 and M2 types. M1 macrophages can secrete pro-inflammatory cytokines such as TNF-α to inhibit tumor growth [11,21,48], whereas M2 macrophages can secrete anti-inflammatory cytokines like TGF-β, promoting tumor growth, angiogenesis, metastasis, and invasion [49,50]. Our results indicate that the expression of M2-related genes initially increases in the coculture system and then significantly decreases upon the application of cucurbitacin I, whereas M1-related genes exhibit the opposite pattern. This altered gene expression profile suggests a dynamic modulation in macrophage polarization toward a more proinflammatory and immune-stimulatory state. Furthermore, our results validate the robust capability of cucurbitacin I to significantly suppress the protumor migratory potential exhibited by M2 macrophages.

Several studies have indicated that cucurbitacin I modulates multiple intracellular cytokines within tumor cells. For example, cucurbitacin I reduces the levels of p-JAK2 and p-STAT3 proteins and decreases the expression of CCND1 and CCNA2 in pancreatic cancer cells [51]. In addition, cucurbitacin I treatment results in the downregulation of MAPK, mTOR, and Akt expression in hepatocellular carcinoma [52]. However, previous studies have predominantly focused on evaluating the effects of cucurbitacin I on tumor cells, demonstrating its role in inhibiting tumor progression through related signaling pathways and cytokines. As a result, limited attention has been directed toward its effects on TAMs and the specific underlying mechanisms. To identify the molecular mechanism underlying the transition of M2-like macrophages to an M1 phenotype induced by cucurbitacin I as well as the differences in gene expression between the untreated and cucurbitacin I-treated groups, we performed RNA sequencing on macrophages from the control group and macrophages treated with cucurbitacin I. The results indicated that *Hmox1*, which was upregulated under coculture/hypoxia conditions and subsequently downregulated following cucurbitacin I treatment, was significantly enriched in various signaling pathways. *Hmox1*, also known as heme oxygenase-1, is involved in heme degradation and implicated in various cellular processes [53]. *Hmox1* is expressed in numerous cell types present in the TME, such as malignant tumor cells, macrophages, dendritic cells, and regulatory T-cells, and contributes to tumor progression through various mechanisms [54,55,56]. Based on single-cell expression data, we observed that, although *Hmox1* was expressed in various cell types, it exhibited significant cell-type specificity, particularly high expression in myeloid cells. Subcluster analysis indicated a significant upregulation of *Hmox1* expression in M2-like macrophages compared with M1-like macrophages. Based on these results, we hypothesized that *Hmox1* acts as one of the potential mechanisms mediating the inhibitory effects of cucurbitacin I on M2 polarization. This hypothesis was validated through a series of experiments, which revealed that both the mRNA and protein levels of *Hmox1* were downregulated in cucurbitacin I-treated M2 macrophages. Therefore, we speculated that Hmox1 acted as one of the mediators for cucurbitacin I’s inhibition of M2 polarization in macrophages. Interestingly, treatment with hemin (an inducer of *Hmox1*) restored the decreased protumor cell migration ability in macrophages treated with cucurbitacin I. It is worth noting that this mechanism may not represent the sole action of cucurbitacin I. Cucurbitacin I, a well-validated JAK2 inhibitor, can suppress JAK2 phosphorylation [51,57]. Additionally, hemin, aside from being an activator of Hmox1, demonstrates the ability to modulate the activity of various transcription factors, such as promoting JAK2 phosphorylation [58]. Competition at the JAK2 level could also be one of the reasons leading to the restoration of macrophage migration ability after cucurbitacin I treatment. Further research is needed to validate the proposed regulatory signals.

In conclusion, our study underscores the multifaceted effects of cucurbitacin I in vitro, particularly in inhibiting tumor cell phenotypes and suppressing the polarization of M2 macrophages. Additionally, we have identified the significant downregulation of the Hmox1 gene in cucurbitacin I treated macrophages. Furthermore, advancements in drug delivery systems have facilitated the development of nanoparticle carriers such as liposomes, polymer nanoparticles, and metal nanoparticles, as well as targeted delivery methods. These carriers and methods enhance the specific delivery of compounds like cucurbitacin I to TAMs, improving their in vivo delivery efficiency or increasing drug concentrations within cancer tissues to minimize damage to healthy tissues, thereby polarizing TAMs towards an anti-tumor phenotype. Combining cucurbitacin I research with these drug delivery systems holds the potential to enhance its application prospects in cancer treatment. This integrated approach allows for more precise delivery of cucurbitacin I to tumor sites, reducing systemic toxicity and improving treatment efficacy. Such a comprehensive strategy is poised to play a significant role in the future of cancer therapy. Therefore, additional studies are needed to further understand the underlying molecular mechanisms in vivo and assess the clinical potential of cucurbitacin I in cancer treatment.

## 4. Materials and Methods

### 4.1. Data Source

We retrieved RNA-seq data and clinical information for 33 cancer types from The Cancer Genome Atlas (TCGA) through the UCSC Xena data portal (https://xenabrowser.net/, accessed on 1 January 2023). Single-cell RNA-seq datasets for breast cancer were obtained from the Broad Institute Single Cell Portal (SCP1039), whereas colon cancer single-cell RNA-seq data were acquired from the Broad Institute Single Cell Portal (SCP1162). The original sequencing data generated for our research are securely archived in the Genome Sequence Archive [59] at the National Genomics Data Center [60], China National Center for Bioinformation, and the Beijing Institute of Genomics, Chinese Academy of Sciences (GSA-Human: HRA005578). These data are publicly accessible at https://ngdc.cncb.ac.cn/gsa-human (accessed on 1 June 2023).

### 4.2. RNA-seq Analysis

A total of 21 macrophage samples were prepared (with three biological replicates per group) for RNA sequencing. Total RNA was isolated using the TRIzol reagent following the manufacturer’s instructions and subsequently resuspended in RNase-free water. The concentration, purity, and integrity of the RNA were assessed by using the RNA Nano 6000 Assay Kit of the Bioanalyzer 2100 system (Agilent Technologies, Santa Clara, CA, USA). The specific methods for library construction and sequencing are described in detail in our previous publication [61].

### 4.3. Differential Expression Analysis

Differential expression analysis was carried out by using the DESeq2 package (version 2.1.28) in R software version 4.2.1. To account for multiple testing, we applied the False Discovery Rate (FDR) correction method. Significantly differentially expressed genes (DEGs) were identified based on the criteria of FDR < 0.05 and an absolute fold change (|FC|) of ≥1.2. The heat maps are drawn by using the “pheatmap” packages, the volcanic plot and Venn diagram are performed using Hiplot website (https://hiplot.cn/, accessed on 1 August 2023).

### 4.4. Functional Enrichment Analysis

DEGs obtained from our study were subjected to functional enrichment analysis by using the KOBAS-intelligence tool [62], accessible at http://kobas.cbi.pku.edu.cn/ (accessed on 1 April 2023). The DEGs were uploaded, and the analysis was performed using default parameters. An interactive visualization tool specifically designed for exploratory data analysis, Hiplot (https://hiplot.cn/ accessed on 1 August 2023), was used to generate a horizontal bar chart visualization based on the relevant parameters and metrics derived from our study.

### 4.5. Single-Cell RNA-Sequencing (scRNA-seq) Analysis

Single-cell RNA-sequencing (scRNA-seq) data were analyzed by using R version 4.0.3 software and R packages, including “dplyr”, “Seurat”, “patchwork”, “sctransform”, “ggplot2”, “SCpubr”, “ggsci”, “limma”, “msigdbr”, “readr”, and so on. Raw data were read using the Read10× function, and then we calculated the mitochondrial gene fraction of every cell so as to filter out cells with a high mitochondrial fraction. Data were normalized using “sctransform” packages, and cell clusters were manually annotated according to known cell marker genes; detailed information for cell marker genes is described in Supplementary Tables S1–S3. Gene set activities within individual clusters of the single-cell dataset were effectively assessed by using the “ssgsea” function from the “GSEABase” package, genes included in esch gene set are listed in Supplementary Table S4.

### 4.6. Reagents and Antibodies

Cucurbitacin I was purchased from TOPSCIENCE (TQ0196) and hemin was purchased from MedChemExpress (HY-19424). The two drugs were dissolved separately in dimethyl sulfoxide (DMSO) to a final concentration of 50 mM. The Hoechst Staining Kit (C1052-1) and The Apoptosis Detection Kit with annexin V-FITC and propidium iodide (C1062L-2) were purchased from Beyotime Biotechnology (Nantong, China). Antibodies against HMOX1 (sc-136960) and CD163 (sc-20066) were purchased from Santa Cruz Biotechnology (Santa Cruz, CA, USA).

### 4.7. Cell Lines and Cell Culture

The human breast cancer cell line MCF7 (cultured in DMEM high glucose medium), the human colorectal cancer cell line HCT116 (cultured in McCoy’s 5a medium), and the human monocyte cell line THP1 (cultured in RPMI-1640 medium) were obtained from the National Collection of Authenticated Cell Cultures; all cells were cultured in complete medium containing 10% fetal bovine serum at 37 °C with 5% CO_2_.

### 4.8. Co-Culture Procedures

The co-culture experiments were performed in six-well plates using Falcon cell culture inserts (Corning, NY, USA). After plating THP1 cells in the upper Transwell chamber, THP1 cells were subjected to stimulation with phorbol 12-myristate 13-acetate (PMA) at a concentration of 100 ng/mL for a duration of 48 h; the PMA-containing medium was removed and fresh culture medium was replaced to continue culturing for 24 h. Next, THP1-derived macrophages were subjected to treatment with different concentrations of cucurbitacin I, according to the previously mentioned experimental grouping. Tumor cells were inoculated into the lower Transwell chamber and grown overnight to allow them to adhere. Inserts containing THP1-derived macrophages were placed into six-well plates containing tumor cells and cells were cocultured for 48 h simultaneously.

### 4.9. Cell Counting Kit-8 (CCK-8) Assay

Tumor cells were initially seeded into 96-well plates at a density of 10,000 cells per well. Subsequently, they were allowed to culture overnight at 37 °C with 5% CO_2_. Following this incubation period, the cells were treated with cucurbitacin I for 24 h. Cell viability was monitored using a CCK8 kit (GK10001, GLPBIO, Montclair, NJ, USA) according to the manufacturer’s instruction; the optical density (OD) value was determined at a wavelength of 450 nm using a Synergy H1 microplate reader (Bio-Teck, Winooski, VT, USA); cell proliferation activity (%) can be calculated by: (OD_treatment-group_ − OD_Blank_)/(OD_control-group_ − OD_Blank_) × 100%.

### 4.10. Scratch Wound-Healing Migration Assay

Tumor cells were seeded into 6-well plates (200,000 cells/well) and cultured overnight at 37 °C with 5% CO_2_; a cell scratch spatula was used to make a scratch in the cell monolayer when the culture reached 80–90% confluence. Digital images were taken at the beginning of the experiment (0 h), which was considered 100%. Tumor cells were incubated with the cucurbitacin I at 0 nM, 1/10 IC50, ¼ IC50, ½ IC50 concentrations, the migration of tumor cell scratch was observed at 12 h, 24 h, and 48 h using an inverted fluorescence microscope (MF53-N, MSHOT, Guangzhou, China). ImageJ was used to analyze the scratched area in the scratch assay quantitatively.

### 4.11. Colony Formation Assay

Tumor cells were cultured in 6-well plates, with or without exposure to cucurbitacin I, at a seeding density of 2500 cells per well. The cells were allowed to grow for a period spanning 7 to 14 days under the specified experimental conditions. Subsequently, the cells underwent a series of steps: they were thoroughly washed with PBS, fixed using a 4% paraformaldehyde solution (P0099, Beyotime Solarbio, China) for 30 min, stained with crystal violet (C8470, Beijing Solarbio, Beijing, China) for 10–15 min, and air dried by inverting the plates onto absorbent paper. Microscopic images of the stained cells were captured and quantified using Image-Pro Plus 6.0 software. The clone formation rate, a measure of cellular colony-forming ability, was calculated as the percentage of clone formations relative to the number of initially inoculated cells. This procedure enabled an assessment of how cucurbitacin I treatment influenced the ability of tumor cells to form colonies.

### 4.12. Quantitative Real-Time PCR Analysis (RT-qPCR)

Total RNA was extracted from the collected samples using the TRIzol method. Inverse transcription was carried out using the Evo M-MLV RT Kit with gDNA Clean for qPCRII (AG11711, Accurate Biotechnology, Changsha, China). To quantify the relative RNA expression levels of target genes, quantitative real-time PCR (RT-qPCR) analysis was performed. The RT-qPCR reaction mixture had a total volume of 20 µL, consisting of 10 µL of 2× SYBR Green Pro Taq HS Premix* (AG11701, Accurate Biotechnology, China), 2 µL of cDNA, 0.4 µL of primers, and 7.6 µL of RNA-free water. The specific primer sequences are detailed in Supplementary Table S5. Normalized fold expression was determined using the 2^−ΔΔCt^ method, and all experiments were conducted in triplicate.

### 4.13. Western Blotting (WB) Analysis

Cell lysis was performed using RIPA lysis buffer (P0013B, Beyotime, China) containing PMSF (ST506, Beyotime, China) and Protease inhibitor cocktail (P1005, Beyotime, China) (200 μL per well of 6-well plates). The cell lysates were then centrifuged at 12,000 rpm, 4 °C, for 5 min following the lysis step on ice. SDS-PAGE Sample Loading Buffer (P0015L, Beyotime, China) was added to the protein extracts and denatured at 95 °C for 5 min. The protein extracts were separated on a sodium dodecyl sulfate polyacrylamide gel (SDS-PAGE), followed by probing with primary and secondary antibodies. Protein bands were detected using the Tanon 4600 Automatic Chemiluminescence Image Analysis System (Tanon, Shanghai, China) and quantified using ImageJ software (ImageJ 1.8.0).

### 4.14. Flow Cytometry Analysis

Cell cycle and apoptosis analysis were conducted using a flow cytometry machine (BD FACS Accuri C6, BD Biosciences, San Jose, CA, USA). The obtained data were subsequently analyzed using FlowJo v9.0 software. The experimental protocols closely adhered to the guidelines specified in the respective kit manuals.

### 4.15. Statistical Analysis

Statistical analyses were conducted by independent T-test (two groups) or one-way ANOVA (multiple groups) analysis using GraphPad 9.4.1 (GraphPad Software Inc., SanDiego, CA, USA), *p*-value < 0.05 was considered statistically significant (* *p* < 0.05, ** *p* < 0.01, *** *p* < 0.001).

## 5. Conclusions

In conclusion, this research presents groundbreaking evidence that cucurbitacin I can efficiently promote the polarization of M2 TAMs towards the M1 phenotype, resulting in the suppression of tumor cell migration. These discoveries hold substantial implications for the advancement of innovative therapeutic approaches and provide a fresh outlook for individuals suffering from cancer.

## Data Availability

The original sequencing data generated for our research are securely archived in the Genome Sequence Archive at the National Genomics Data Center, China National Center for Bioinformation, and the Beijing Institute of Genomics, Chinese Academy of Sciences (GSA-Human: HRA005578). These data are publicly accessible at https://ngdc.cncb.ac.cn/gsa-human accessed on 1 August 2023.

## Supplementary Materials

The supporting information can be downloaded at: https://www.mdpi.com/article/10.3390/ijms242115920/s1.
