# Supplementary material for: Cucurbitacin I Reverses Tumor-Associated Macrophage Polarization to Affect Cancer Cell Metastasis
Source: Int J Mol Sci. 2023 Nov 2;24(21):15920. doi: 10.3390/ijms242115920 (PMC10650020; doi:10.3390/ijms242115920)
Supplement: Supplementary file 1 [file ijms-24-15920-s001.zip › Supplementary_Material-Figures.pdf]

Supplementary Material

Supplementary Figures

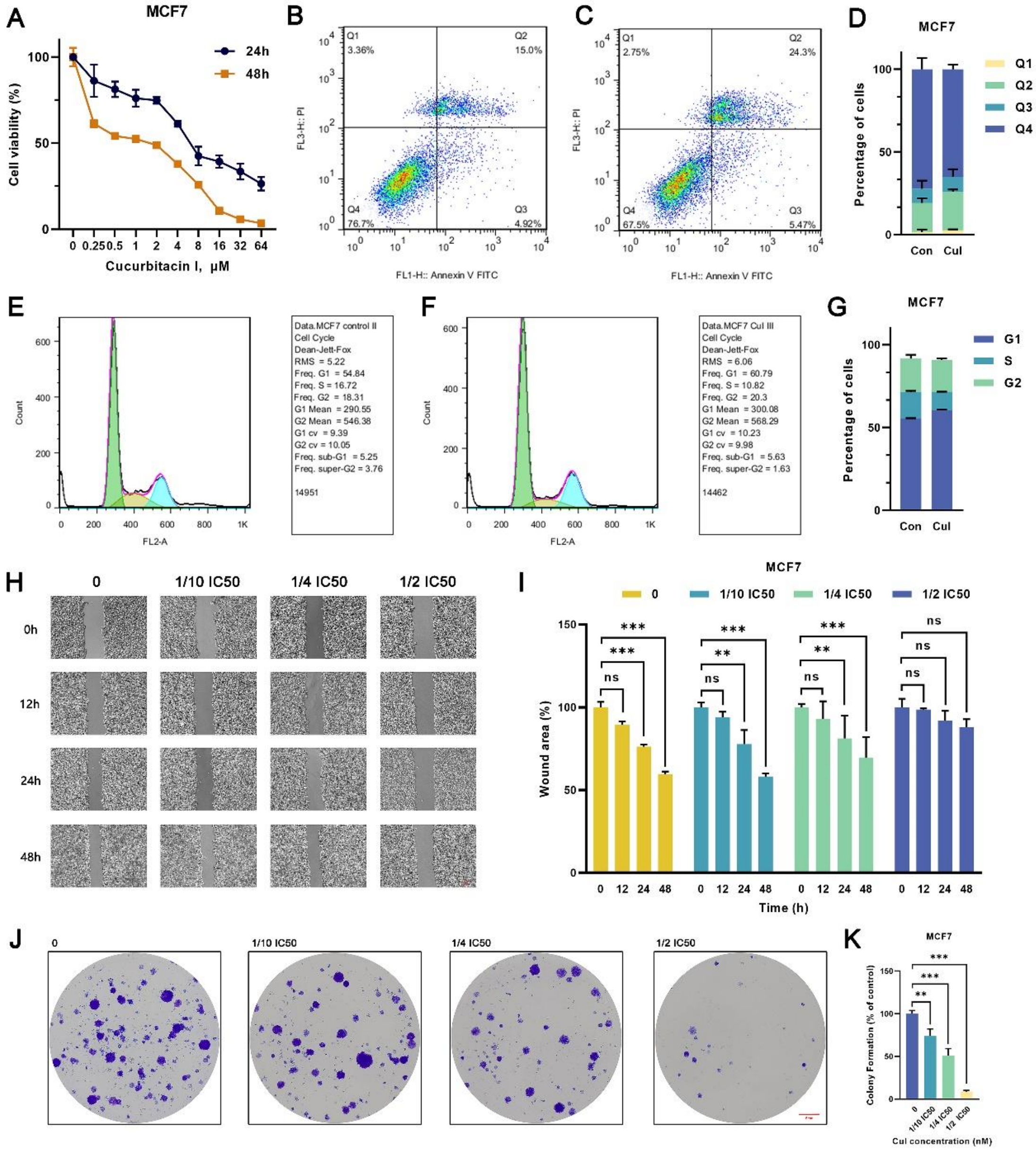

Supplementary Figure S1. Cucurbitacin I has an inhibitory effect on BRCA cell phenotype. **A.** HCT116 cells were treated with cucurbitacin I (0, 0.25, 0.5, 1, 2, 4, 8, 16, 32, 64μM) for 24 h and 48 h, and cell viability (%) was assayed. **B-D.** Representative flow-cytometric plots and its quantitative results (D) depicting the apoptotic characteristics of MCF7 cells treated with cucurbitacin I (500nM) (C) compared with non-treated (B). **E-G.** Flow cytometry indicated cucurbitacin I resulted in changes

in cell proportions at the G1/S phase in MCF7 cells cultured in the absence (E) and presence (F) of 500nM cucurbitacin I. In the flow cytometry plot, the green peak represents cells in the G1 phase, the yellow peak represents cells in the S phase, and the blue peak represents cells in the G2 phase. **H-I.** Scratch assay of HCT116 cells with cucurbitacin I treatment (0nM, 1/10 IC50, 1/4 IC50, 1/2 IC50) in different concentrations and results of wound area (%) were calculated in (I). Scale bar represents 100µm. **J-K.** The colony-formation assay of HCT116 cells with cucurbitacin I treatment at different concentrations was performed and colony numbers were shown in (K). Scale bar represents 5mm. values are mean ± SD., n = 3 biologically independent samples. *, $p < 0.05$, **, $p < 0.01$, ***, $p < 0.001$, ns, no significance.

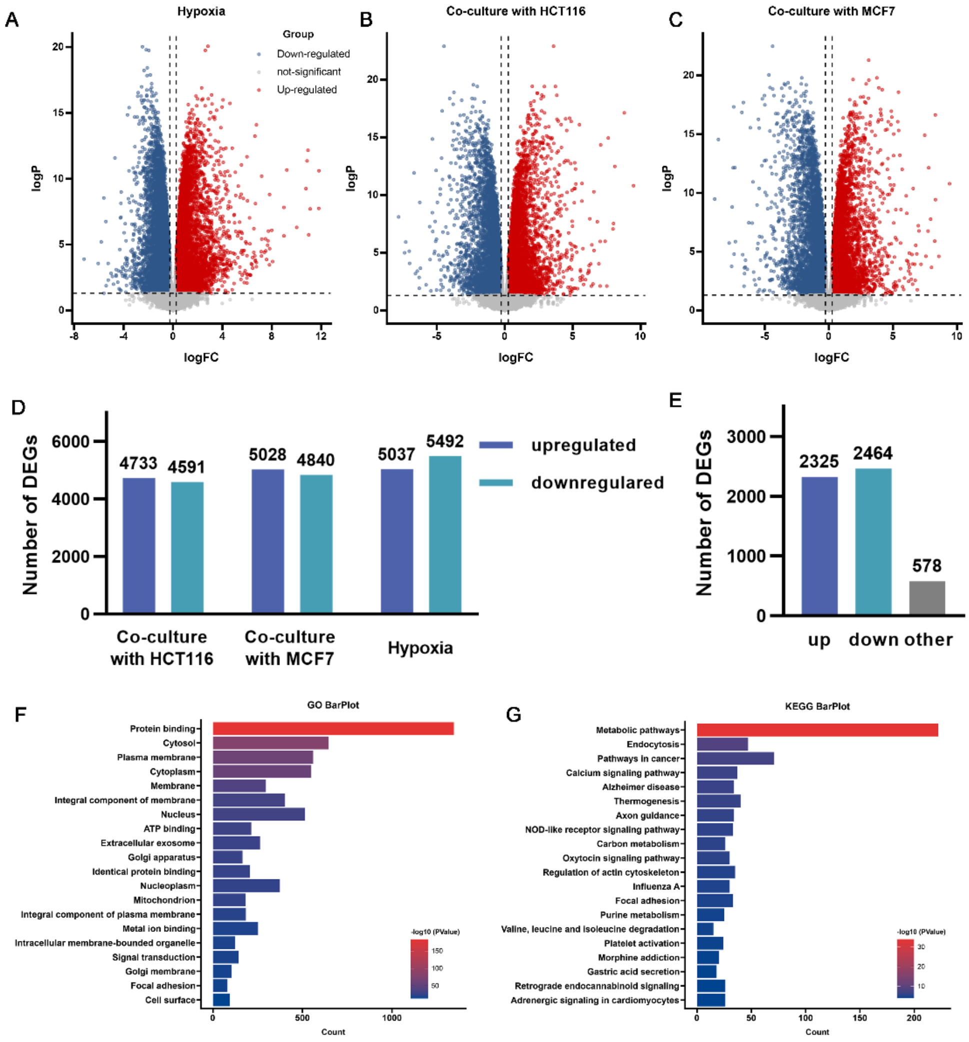

Supplementary Figure S2. Transcriptome Data analysis of Macrophage under Hypoxia or in co-culture systems. **A-C.** Volcano plot showing the DEGs in different treatment- macrophages (hypoxia (A), co-cultured with HCT116 (B) or MCF7 (C) cells vs non-treated). Difference between percentage of cells expressed in two clusters was plotted against log fold change of average expressions. Each red dot denotes an up-regulated gene. Each blue dot denotes a down-regulated gene. Each grey dot denotes a non-significant gene. **D.** The histogram showed the specific number of upregulated and downregulated genes in each group. **E.** The histogram showed the specific number of upregulated

and downregulated genes in the three overlapping genes above. **F-G.** GO (F) and KEGG (G) term analysis of downregulated DEGs from overlapping genes in the above groups (A-C) and the top twenty terms were represented. adjusted P ⩽ 0.05.

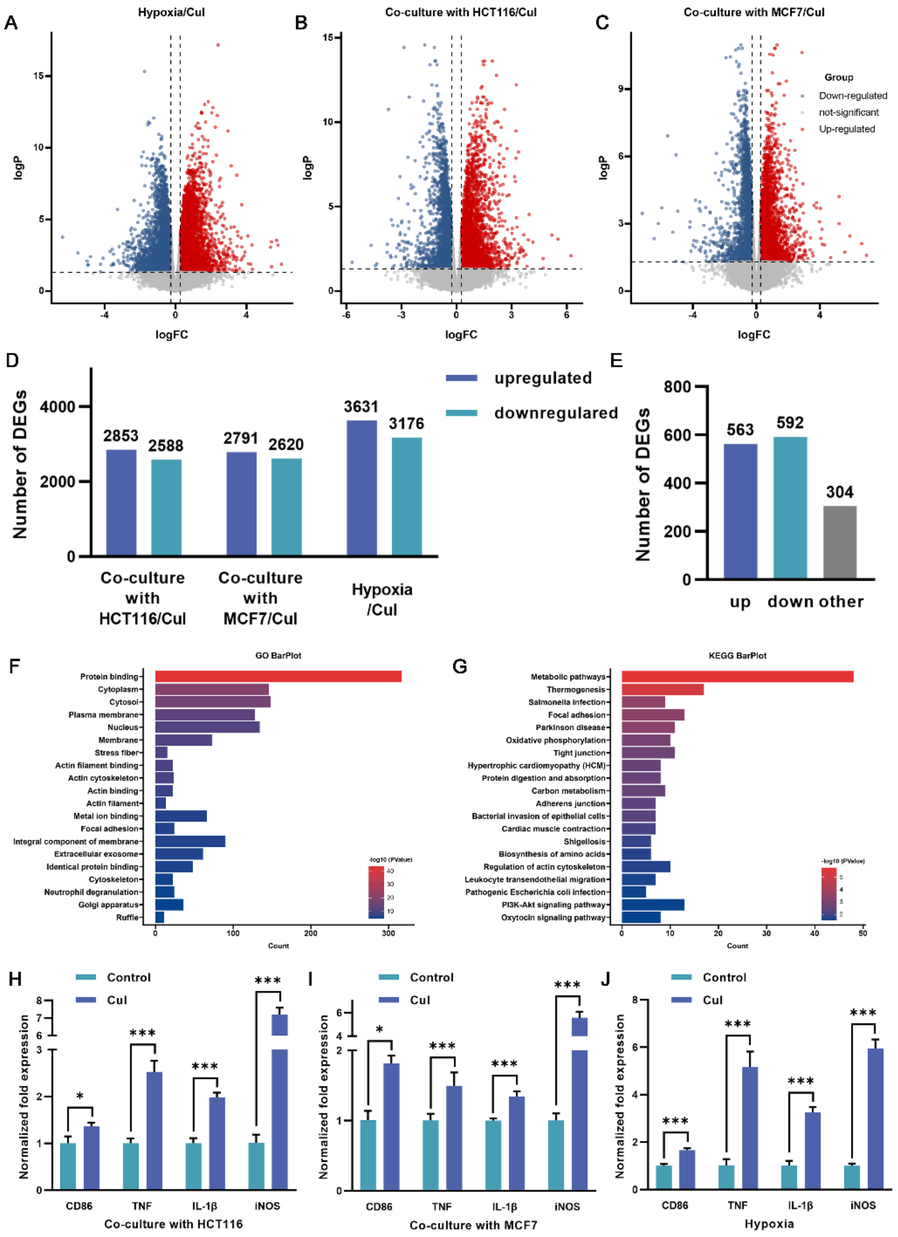

Supplementary Figure S3. Transcriptome Data analysis of Macrophage being treated with cucurbitacin I under hypoxia or in co-culture systems. **A-C.** Volcano plot showing the DEGs in different treatment- macrophages (hypoxia (A), co-cultured with HCT116 (B) or MCF7 (C) cells vs non-treated). Difference between percentage of cells expressed in two clusters was plotted against log fold change of average expressions. Each red dot denotes an up-regulated gene. Each blue dot denotes a down-regulated gene. Each grey dot denotes a non-significant gene. (D) The histogram showed the specific number of upregulated and downregulated genes in each group. **E.** The histogram showed the specific number of upregulated and downregulated genes in the three overlapping genes above. **F-G.** GO (F) and KEGG (G) term analysis of downregulated DEGs from

overlapping genes in the above groups (A-C) and the top twenty terms were represented. adjusted P ⩽ 0.05. **H-J.** cucurbitacin I upregulated mRNA level of M1-related genes in macrophages, cultured in three systems, namely co-cultured with HCT116 cells (H), MCF7 cells (I), or under hypoxia (J).

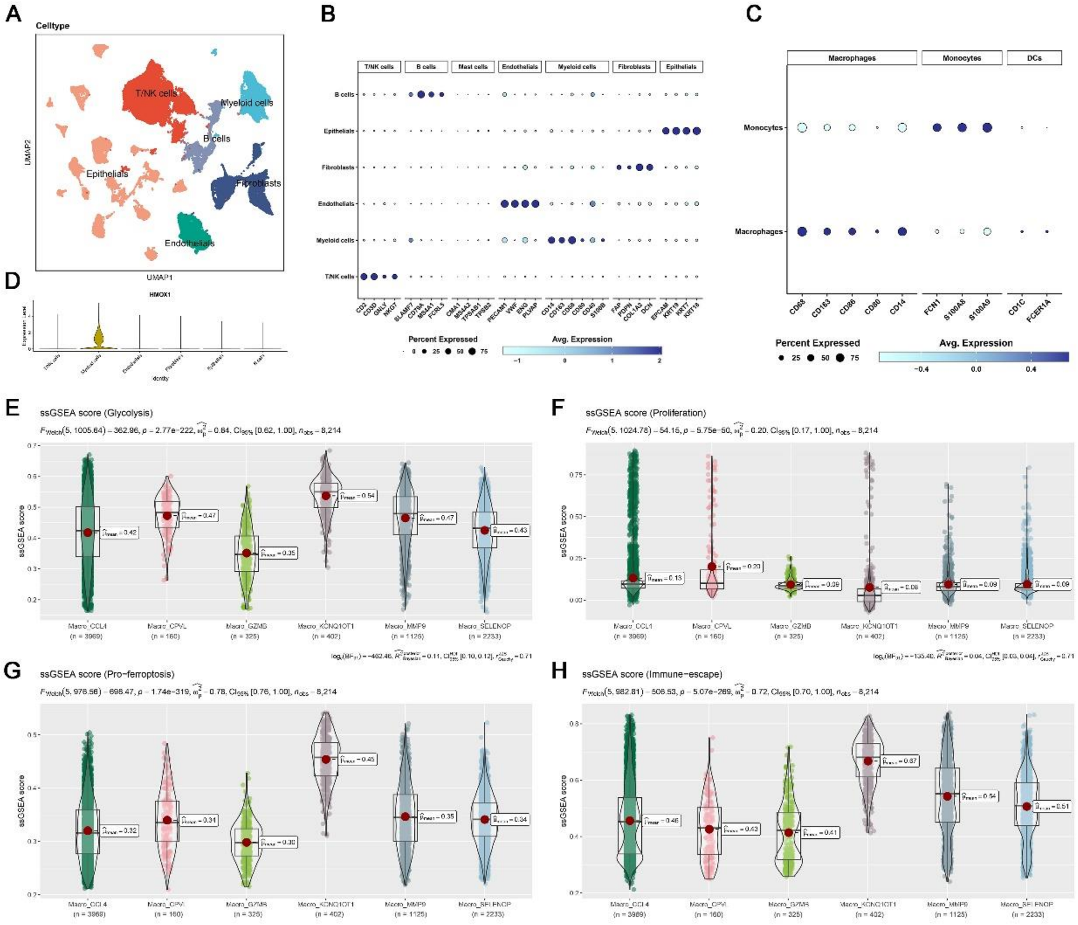

Supplementary Figure S4. Single-cell cluster analysis and ssGSEA scores in BRCA. **A.** UMAP dimension 1 and 2 plot showing different cell populations, colored by their 6 major cell types. B. Dot plots of marker gene expression (x-axis) across BRCA cell clusters identified (y-axis). C. Dot plots of marker gene expression (x-axis) across macrophages and monocytes separated from myeloid cells (y-axis). For B and C, the cell type corresponding to the marker gene is displayed at the top. Average expression levels were represented by color, and dot size displayed the percent expressed levels. **D.** Violin plot displayed that *Hmox1* was significantly enriched in myeloid cells. **E-H.** Boxplot displaying ssGSEA score in six macrophage-subclusters from scRNA-seq of BRCA. Of which, the

ssGSEA score involved in glycolysis (E), proliferation (F), pro-ferroptosis (G), and immune-escape(H) was calculated, boxes show the median and whiskers indicate the 95th and 5th percentiles.

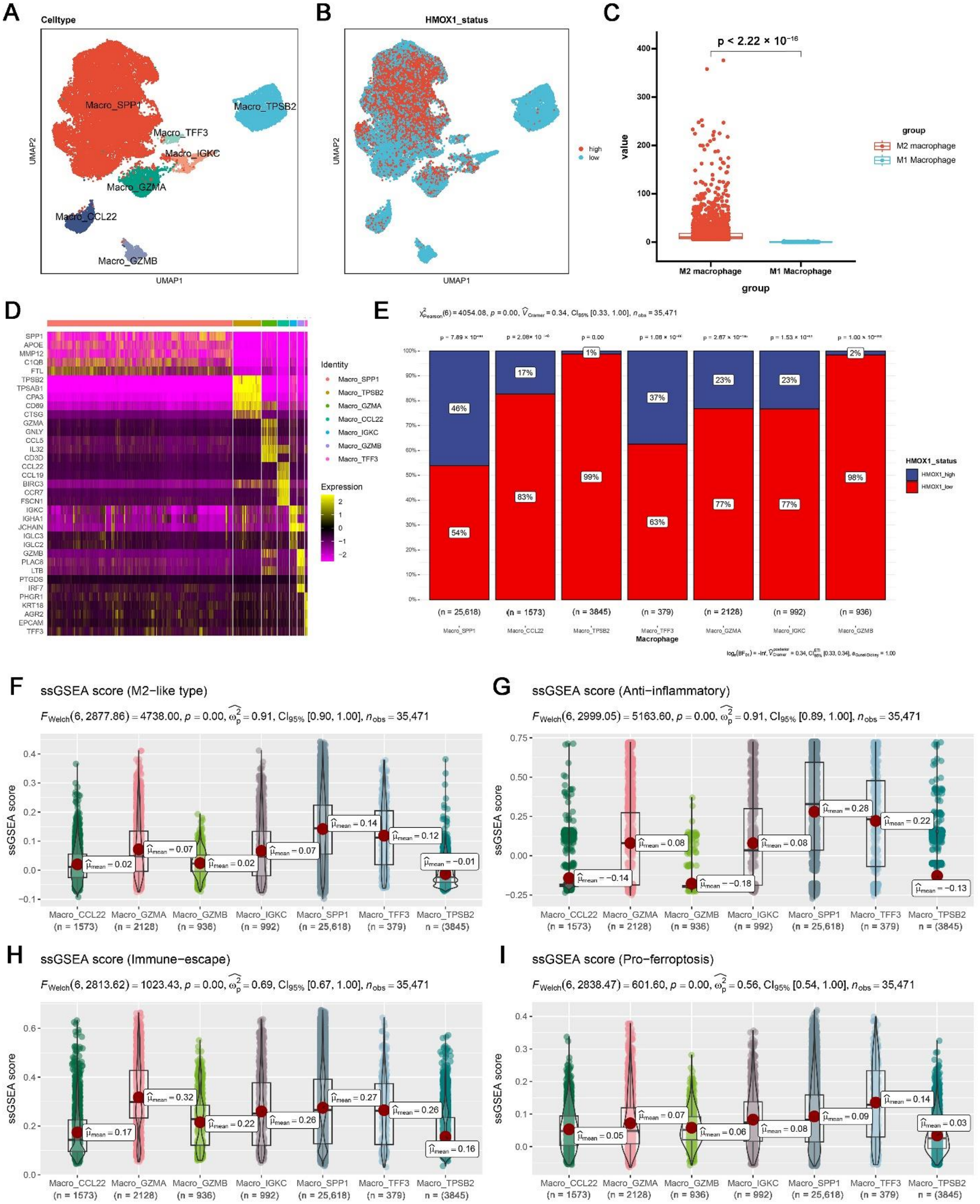

Supplementary Figure S5. Single-cell analysis of *Hmox1* expression profile reveals the relationship between *Hmox1* and cancer-related processes in CRC. **A.** The UMAP visualization of macrophages from patients with colorectal cancer, showing the formation of 7 main clusters shown in different colors. The functional description of each cluster is determined by the gene expression characteristics of each cluster. **B.** The UMAP visualization of macrophages, with each cell colored based on the relative normalized expression levels of Hmox1. **C.** Expression levels of Hmox1 in M2 macrophages in comparison with M1 macrophages from CRC tumor samples, boxes show the median and whiskers indicate the 95th and 5th percentiles. **D.** Heatmap of marker gene expression (y-axis) across CRC macrophages clusters identified (x-axis). **E.** Histogram displays cell percentage of high and low *Hmox1* expression groups in five macrophage subclusters, respectively. **F-I.** Boxplot displaying ssGSEA score in six macrophage-subclusters from scRNA-seq of CRC. Of which, ssGSEA score involved in M2-like type (F), anti-inflammatory (G), immune-escape (H), pro-ferroptosis (I), boxes show the median and whiskers indicate the 95th and 5th percentiles.

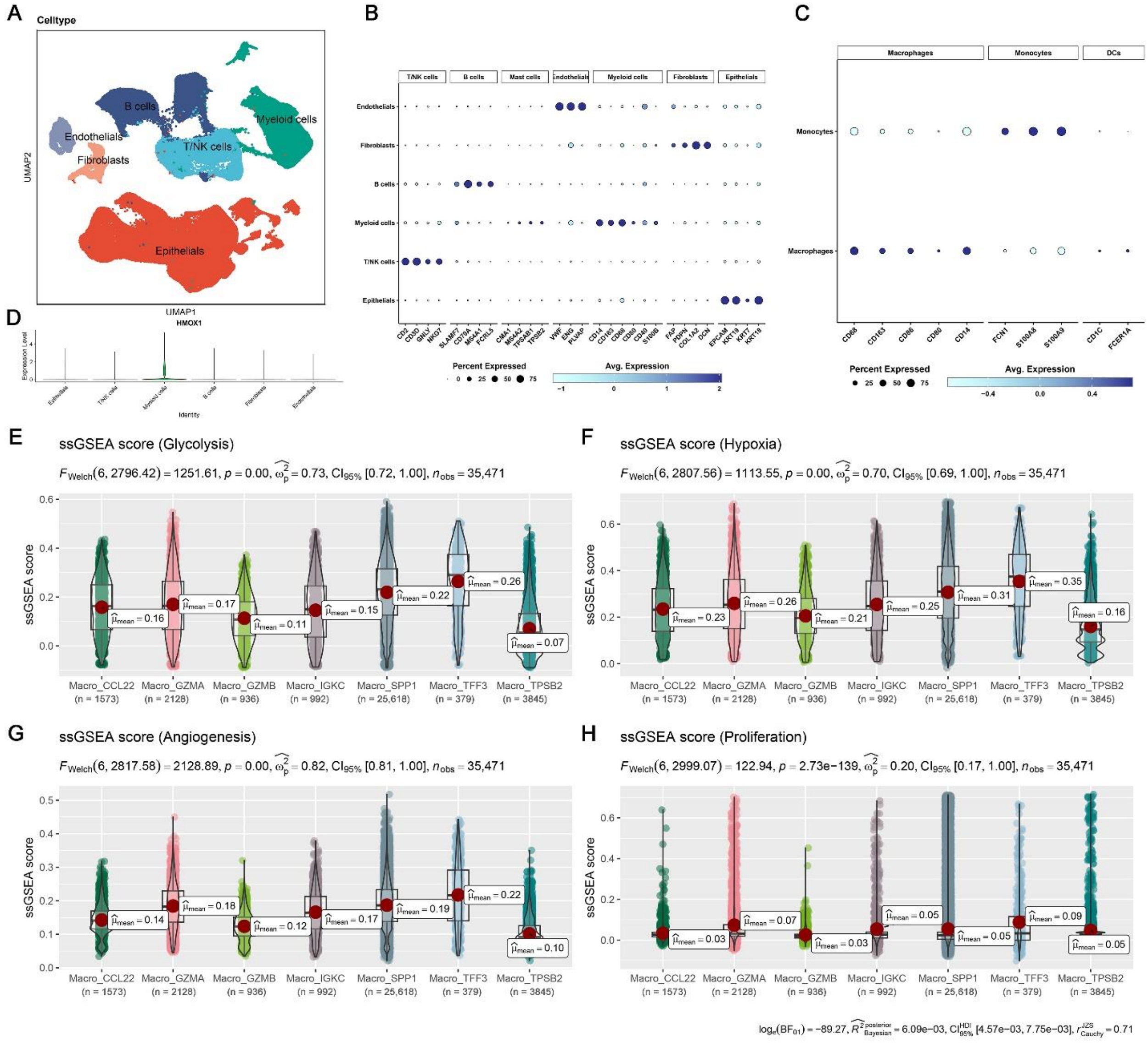

Supplementary Figure S6. Single-cell cluster analysis and ssGSEA scores in CRC. **A.** UMAP dimension 1 and 2 plot showing different cell populations, colored by their 6 major cell types. **B.** Dot plots of marker gene expression (x-axis) across CRC cell clusters identified (y-axis). **C.** Dot plots of marker gene expression (x-axis) across macrophages and monocytes separated from myeloid cells (y-axis). For B and C, the cell type corresponding to the marker gene is displayed at the top. Average expression levels were represented by color, and dot size displayed the percent expressed levels. **D.** Violin plot displayed that *Hmox1* was significantly enriched in myeloid cells. **E-H.** Boxplot displaying ssGSEA score in six macrophage-subcluster from scRNA-seq of CRC. Of which, ssGSEA

score involved in glycolysis (E), hypoxia (F), angiogenesis (G), and proliferation (H) was calculated, boxes show the median and whiskers indicate the 95th and 5th percentiles.

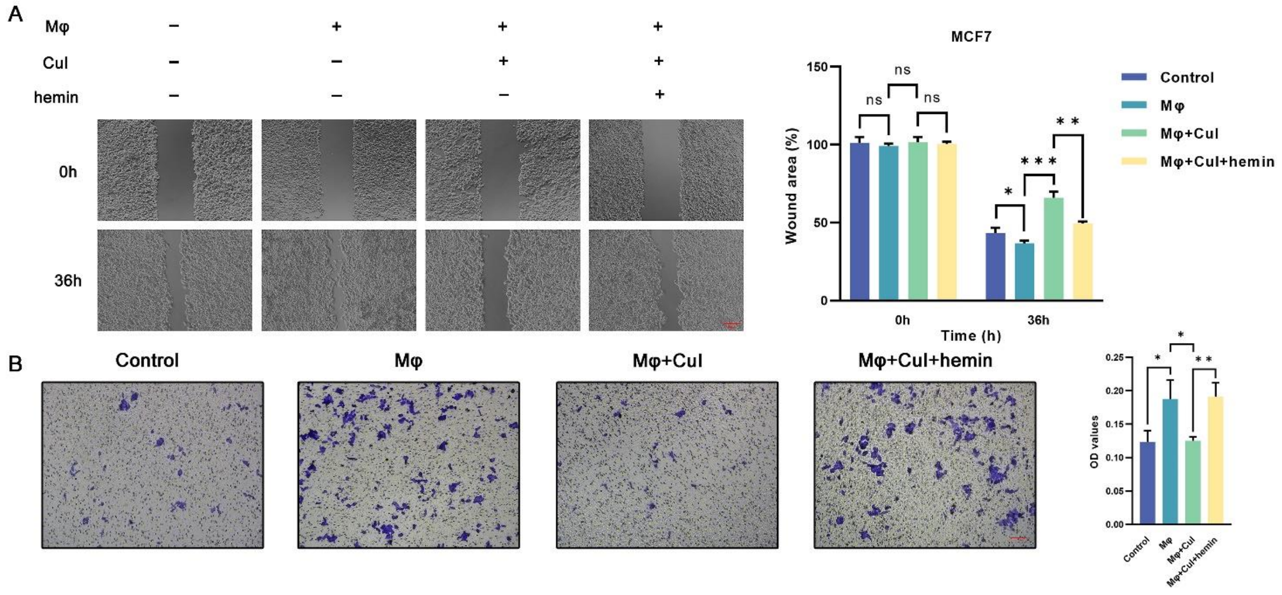

Supplementary Figure S7. Cucurbitacin I augments the tumoricidal activity by influencing the polarization of macrophages, which could be reversed by hemin. **A-B.** Cell migration and invasion capacity of MCF7 alone or co-cultured with macrophages (alone, cucurbitacin I-treated macrophages or both cucurbitacin I and hemin–treated macrophages) was determined by the wound healing assay and transwell coculture system, respectively. Scale bar represents 100µm (**A**) and 50µm(**B**). Data are presented as the mean ± SD from three independent experiments. *, $p < 0.05$, **, $p < 0.01$, ***, $p < 0.001$, ns, no significance.
